# Management of Cervical Spine Fractures: A Literature Review

**DOI:** 10.7759/cureus.14418

**Published:** 2021-04-11

**Authors:** Mohammad Waseem Beeharry, Komal Moqeem, Mujeeb U Rohilla

**Affiliations:** 1 Trauma and Orthopaedics, Barts Health NHS Trust, London, GBR; 2 Emergency Department, Royal Surrey County Hospital, Guildford, GBR; 3 Trauma and Orthopaedics, Milton Keynes University Hospital, Milton Keynes, GBR

**Keywords:** cervical spine, c-spine, cervical trauma, cervical spine fracture

## Abstract

The unique anatomy and flexibility of the cervical spine predispose it to a risk of injury. Trauma to the cervical spine encompasses a wide range of injuries from minor muscular strains to life-threatening fracture-dislocations associated with spinal cord lesions. Initial assessment and management should follow the Advanced Trauma Life Support (ATLS) protocols with adequate protection of the cervical spine through triple immobilisation to prevent any unnecessary movement, which can make the patient susceptible to further neurological injuries. Although the presence of cervical spine injury is very often overt, reliance on clinical examination alone is sometimes not sufficient and potentially requires further imaging. Clinical decision rules such as the Canadian C-Spine Rule are frequently used to risk-stratify patients needing radiography. The level of cervical spine instability and knowledge of their unique classification systems is of paramount importance and assists in the decision-making process to guide definitive management. In this review, we also propose an algorithm to aid the initial management of a patient with suspected cervical spine injury in the emergency department.

## Introduction and background

Cervical spine (C-spine) trauma constitutes a variety of injuries ranging from relatively mild ligamentous and muscular strains to fractures and dislocations of the bony vertebrae, which can result in significant spinal cord injuries (SCIs). C-spine injuries account for half of all spinal injuries, with approximately 500-600 people enduring acute traumatic SCIs every year in the United Kingdom [1]. Specialised ligamentous and osseous anatomy provides the C-spine with great range of motion but also makes it more susceptible to injury. Varying underlying mechanisms such as hyperflexion, hyperextension, axial loading, rotational and distraction forces predispose the C-spine to injuries [2].

C-spine injuries are sustained following low-energy incidents such as a simple innocuous fall to higher energy traumas, such as in motor vehicle accidents. Passias et al. showed that motor vehicle accidents were the most prevalent cause in the United States, responsible for 29.3% of C-spine fractures and most frequently occurring at the C2 (32.0%) and C7 (20.9%) levels [3]. As the full extent of the injury may not be apparent initially, all patients with suspected C-spine trauma should be assessed systematically using a standardised approach to improve patient outcomes. If not managed accordingly, C-spine injuries can eventually lead to significant morbidity and mortality with numerous functional and psychosocial ramifications.

## Review

Initial assessment of suspected C-spine fracture

Patients with suspected C-spine fracture are invariably managed in the pre-hospital setting with the application of a rigid brace to the C-spine (hard collar) in a neutral position to achieve cephalic immobilisation and keep the spinal column ‘in-line’ in order to prevent undue movement.

On presentation to the emergency department, any suspected C-spine fracture patient should be assumed to have a C-spine injury until proven otherwise. A detailed history, from either the emergency crew or the conscious patient, is of paramount importance and should be solicited whenever possible prior to clinical examination. The history should aim to elicit the type of trauma (high energy v/s low energy), mechanism of injury, and the presence, onset and progression of any neurological or physical symptoms including consideration of any factors that may impair the patient’s ability to recall the sequence of events.

The goal of the initial assessment of suspected C-spine fractures is the prompt recognition and identification of the primary injury and adequate protection of the C-spine to prevent subsequent neurological deficit. While unconscious patients are primarily assessed using trauma CT series (CT of the C-spine, chest, abdomen and pelvis), initial assessment of the conscious, suspected C-spine fracture patient should be performed using a prioritising sequence based on the Advanced Trauma Life Support (ATLS) protocols. The ATLS algorithm begins with maintenance of the airway with restriction and stabilisation of the C-spine using triple immobilisation technique with a hard collar, sandbags, head blocks and tape anchored to the trolley.

Once the C-spine and airway has been secured, next steps include assessment of breathing for adequate oxygenation followed by circulation for satisfactory perfusion. Systemic hypotension due to haemorrhagic or neurogenic shock is often a complication and sign of SCIs [4]. This should be managed promptly with aggressive fluid resuscitation to maintain end-organ perfusion. A detailed assessment of any neurological abnormality, in accordance with the American Spinal Injury Association (ASIA) classification, should follow. This includes sensory examination (light touch and pin prick) to assess for paraesthesia, muscle strength grading for any limb weakness, and normal and pathological deep tendon reflex testing of the biceps, triceps and brachioradialis [5]. SCIs account for approximately 15% of cases with a spinal column fracture or dislocation [1] and can have debilitating consequences. Hence, these injuries must be quickly and reliably identified to provide safe, effective and timely intervention.

**Figure 1:**
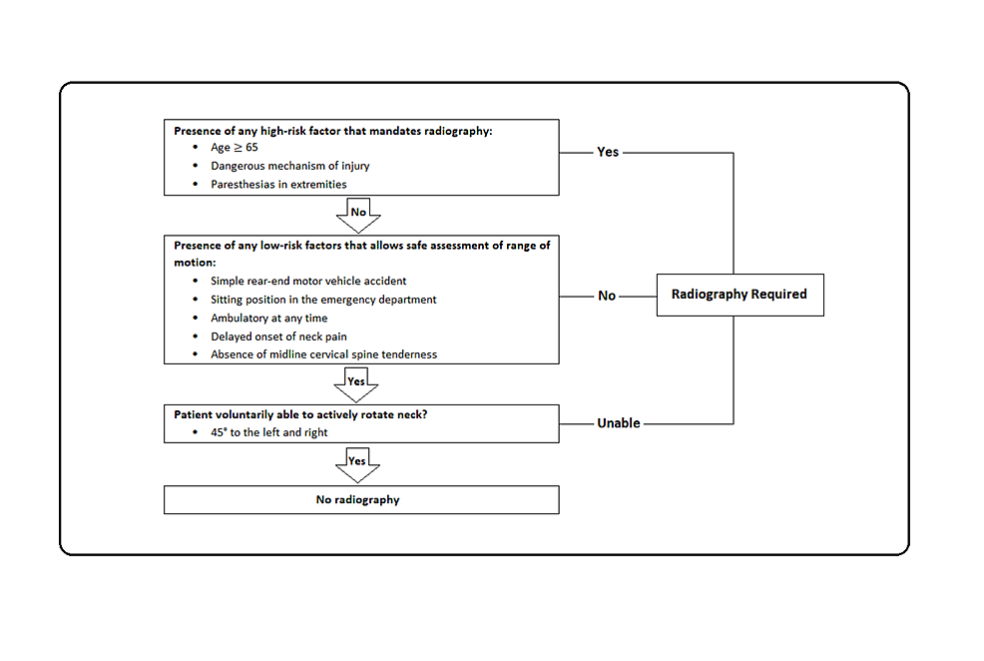
The Canadian C-Spine Rule algorithm for patients who are alert (GCS score of 15) and in stable condition with suspected cervical spine injury to guide further imaging. [6] GCS, Glasgow Coma Scale

Reliance on clinical examination of the C-spine alone is not sufficient to diagnose any secondary or unrecognised injuries to the C-spine, and imaging is required to allow identification of further clinically relevant injuries. In accordance with the National Institute for Health and Care Excellence (NICE) guidelines, suspected cervical trauma patients in hospital are risk-stratified for cervical spinal injury using the Canadian C-Spine Rule (CCR) [6]. The CCR, derived by Stiell et al. in 2001, identifies trauma patients requiring diagnostic imaging based on three high-risk criteria, five low-risk criteria and the ability of patients to rotate their necks (Figure 1) [6]. This set of clinical algorithm has shown high sensitivity in the range of 90% to 100% in several large prospective cross-sectional studies [7].

Radiographs used in the CCR studies included plain films, but imaging of the C-spine has since evolved from plain films to CT scans. CT scan not only provides good visualisation, especially of the transitional zones (craniocervical and cervicothoracic) as compared to standard radiographs, but also has a high sensitivity of about 87.5% for any C-spine lesion and sensitivity of about 100% for any unstable lesion [7].

Initial management of a patient with C-spine fractures

The management of C-spine fractures is guided by the severity of the fracture. The urgency of treatment is dependent on life-threatening airway, respiratory or circulatory compromise along with the presence of a neurological lesion and/or instability. Thus, appropriate initial management plays a crucial role in dictating the long-term prognosis of patients with C-spine fracture.

Patients with C-spine fracture require early and effective analgesia to control pain in the acute setting. The NICE guidelines advocate the use of intravenous morphine as the first-line analgesic, wherever appropriate, or intra-nasal diamorphine or ketamine if intravenous access has not been established [1]. The main focus, however, remains stabilisation of the C-spine as per the ATLS protocol to protect the spinal cord by triple immobilisation of neck movements with a hard collar, sandbags and tape anchored to the trolley. Hard collars must incorporate the chin, occiput or forehead, and they restrict flexion/extension by 20-25% [8]. A study conducted by Askins and Eismont has shown that the NecLoc® cervical collar was more effective than the Miami-J or Philadelphia collar in restricting cervical motion in flexion, rotation, extension and lateral tilt [9].

The application of a cervical collar while a decision is being made is not without complications, which include increase in cerebrospinal fluid pressure, skin ulceration, reduced tidal volume and dysphagia [10]. Additionally, immobilisation of unstable C-spine fractures with rigid cervical collar may prove to be suboptimal as there is evidence to show that such collars do not restrict the displacement of unstable cervical injuries [11]. Cervicothoracic orthoses such as halothoracic bracing can provide more stabilisation by restricting flexion and extension by 70-80%, rotation by 60-70% and lateral flexion by 60% [8].

C-spine fractures can often result in anatomic displacement and instability, which is described as the loss of ability of the spine under physiological loads to maintain its pattern of displacement [12]. A higher degree of instability translates into a greater likelihood of neurological disability secondary to SCIs. Bonner and Smith describe an SCI as ‘damage to the spinal cord caused by an insult resulting in the transient or permanent loss of usual spinal motor, sensory and autonomic function’ [13]. C-spine injuries have the highest reported mortality rate of all spinal injuries, often as a result of an increased incidence of SCIs [7]. Timely and pertinent interventions can decrease adverse outcomes by decreasing the probability of neurological sequalae.

**Figure 2:**
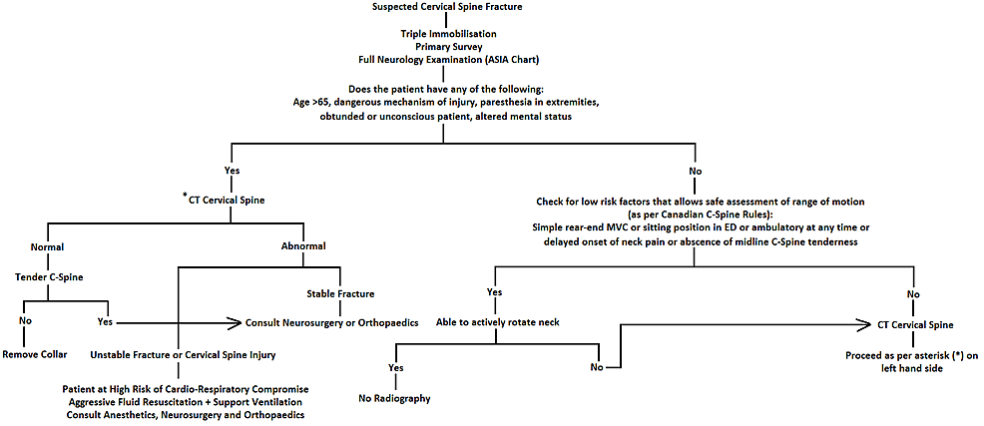
Algorithm for the management of suspected cervical spinal fractures in the emergency department.

Patients with cervical injuries are particularly at risk of cardiorespiratory compromise. High cervical injuries (C1 to C5) are especially prone to deterioration, which can lead to respiratory depression and carbon dioxide retention as a result of diaphragmatic function insufficiency. Respiratory compromise, which can develop within the first 5 days secondary to ascending oedema, is the principal cause of death in patients with SCI [14]. Therapeutic action of patients with neurologic compromise should aim to maintain a mean arterial pressure (MAP) of 85-90 mmHg for up to seven days following injury by either aggressive intravenous fluid resuscitation or inotropic agents if the MAP is not maintained by fluids alone. Ventilation function should be supported to prevent hypoxemia [5]. It is, therefore, not uncommon for high-cord injured patients to be managed with intubation or tracheostomy in the acute setting. Such patients are ultimately optimally managed in the intensive care unit for continuous monitoring. Here, we propose an algorithm (Figure 2) to assist the initial management of the patient with suspected C-spine injury in the emergency department.

Definitive treatment focusing on principles of management

The definitive management of C-spine fractures is primarily guided by the grade of instability conferred by the injury [12]. Any traumatic spinal injury that disrupts the biomechanical stability of the spine can potentially result in both neurological and musculoskeletal injuries that together impact the functional status of the patient [15]. The principal aim of definitive management of C-spine fractures is to minimise the resulting functional disability - achieved either operatively or non-operatively.

Unique classification systems are available for fractures occurring in different regions of the C-spine to assist in the decision-making process regarding the most appropriate definitive management. The C-spine can be viewed as two distinct regions owing to their markedly different anatomical structures - the upper C-spine (occiput to C2) and the subaxial or lower C-spine (C3-7).

The upper C-spine has a distinct set of articulations enabled by the highly specialised atlas (C1) and axis (C2), which provides a great deal of mobility for the skull. This highly mobile region derives its stability from ligaments, and consequently surgical interventions are frequently required in fracture patterns that disrupt ligamentous integrity.

Fracture to the base of the skull or occipital condylar fractures can be classified using the Anderson and Montesano classification system [16]. Type I (impacted fracture of the occipital condyle) and type II fractures (basilar skull fracture including an occipital condyle fracture) are deemed stable and can be managed non-operatively using a semi-rigid or rigid cervical collar [17]. Type III fractures are avulsion fractures of the occipital condyle that can potentially be unstable due to the loss of integrity of the alar ligament that can potentially lead to craniocervical dissociation (CCD) [16]. The craniocervical joint complex consists of structurally important osseous and ligamentous complexes that stabilise the skull base to the spine, which, when disrupted, is indicative of a severe and life-threatening instability. However, CCD is frequently fatal as it results in significant neurological injury to the brainstem [15]. In survivors of CCD who arrive in the emergency department, non-operative management is usually not adequate as significant ligamentous disruption cannot heal even with prolonged external immobilisation. A halo vest can be provisionally used to stabilise the craniocervical junction until definitive surgery can be performed. Operative stabilisation involves rigid posterior segmental stabilisation with instrumentation from the occiput to at least C2 after restoration of alignment [17].

Atlas (C1) fractures account for 2% to 13% of all C-spine injuries. Although several fracture classification systems for atlas fractures are available (e.g., Jefferson, Landells and Gehweiler classifications), the degree of instability and thus requirement for surgery is determined by the integrity of the transverse ligament of the atlas, which is key in providing stability to the C1-C2 region. Dickman et al. classified transverse ligamentous injuries of the atlas into two types [18]. Type I corresponds to intra-ligamentous tears and is surgically managed with C1-C2 fusions, while type II involves a bony avulsion at the tubercle on C1 lateral mass and is treated non-operatively in a halo vest with a 75% chance of success. Stable atlas fracture patterns where the transverse ligament is intact can be managed in a hard collar [19].

Axis (C2) fractures include odontoid peg fractures and traumatic spondylolisthesis of the axis (Hangman’s fracture). Anderson and D’Alonzo classified odontoid peg fractures into three subtypes. Type II fractures through the waist of the odonotoid peg have a high rate of non-union, which occurs as a result of disruption to the blood supply. These fractures are subsequently managed operatively with techniques such as posterior C1-C2 fusion or anterior odontoid screw osteosynthesis. Type I (oblique fracture through the odontoid tip) and type III (fracture through the body) injuries can be managed with a cervical collar [20]. Hangman’s fracture, classified using Levine and Edwards classifications, is based on the mechanism of injury. Surgical treatment of Hangman’s fracture is rare and can be managed non-operatively with a hard collar or closed reduction followed by halo immobilisation in most cases [17].

In the lower or subaxial C-spine (C3-7), the ligamentous and bony structures play an equal role in stability [15]. Definitive management can be guided by the AO Spine classification system, which consists of three main categories: compression injury (type A), disruptions of either the anterior or posterior tension bands (type B) and disruption to both the anterior and posterior tension band with translatory instability (type C) [21]. Type B (further subclassified into types B1, B2 and B3) and type C fractures are unstable injuries requiring operative management [7]. Stable type A compression injuries with intact posterior ligament and no significant kyphosis can be managed non-operatively with external immobilisation [22].

## Conclusions

The priority in every patient with suspected C-spine injury is to stabilise the C-spine and prevent undue movement which can cause further neurological injury using triple immobilisation as per the ATLS algorithm. Cardiorespiratory compromise, which can arise as a result of spinal shock, should be addressed and managed, while intensive care input must be sought wherever necessary. Urgent reduction of the fracture-dislocation and surgical decompression of the spinal cord may be necessitated to improve neurological outcomes. Definitive management of C-spine injuries is guided by the degree of instability of the fractures and can be addressed conservatively or operatively. Surgical management aims to achieve a balanced stable spine to prevent painful deformity, preservation and improvement of neurological function and facilitation of the rehabilitation process by enabling early mobilisation. The benefits of surgery must be weighed against its potential risks on a case-by-case basis.
